# Supplementary material for: Molecular Characterization of a B Cell Adaptor for Phosphoinositide 3-Kinase Homolog in Lamprey (Lampetra japonica) and Its Function in the Immune Response
Source: Int J Mol Sci. 2022 Nov 21;23(22):14449. doi: 10.3390/ijms232214449 (PMC9695028; doi:10.3390/ijms232214449)
Supplement: Supplementary file 1 [file ijms-23-14449-s001.zip › ijms-2035677-supplementary.pdf]

Supplementary Material

Supplementary Table S1. The BCAP and BANK1 protein accession numbers in all examined species

Species	Symbol	Accession No.	Species	Symbol	Accession No.
**Human (*Homo sapiens*)**	BCAP	NP_689522	**Mouse (*Mus musculus*)**	BCAP	NP_113553
	BANK1	NP_060405		BANK1	NP_001028522
**Domestic cat (*Felis catus*)**	BCAP	XP_023096562	**Cow (*Bos taurus*)**	BCAP	XP_002698457
	BANK1	XP_023108677		BANK1	XP_002688165
**Soft-shelled turtle (*Pelodiscus sinensis*)**	BCAP	XP_025037072	**American alligator (*Alligator mississippiensis*)**	BCAP	XP_014460564
	BANK1	XP_006126969		BANK1	XP_019343599
**Chinese alligator (*Alligator sinensis*)**	BCAP	XP_014380105	**Green anole (*Anolis carolinensis*)**	BCAP	XP_003223179
	BANK1	XP_014380105		BANK1	XP_008116471
**Wall lizard (*Gekko japonicus*)**	BCAP	XP_015281369	**Burrowing owl (*Athene cunicularia*)**	BCAP	XP_026706762
	BANK1	XP_015261254		BANK1	XP_026703736
**Mallard (*Anas platyrhynchos*)**	BCAP	XP_005025238	**Bengalese finch (*Lonchura striata*)**	BCAP	XP_021403783
	BANK1	XP_027311808		BANK1	XP_021387118
**Chicken (*Gallus gallus*)**	BCAP	NP_989681	**African clawed frog (*Xenopus laevis*)**	BCAP	XP_018080858
	BANK1	XP_015141103		BANK1	XP_041446622
**Tropical Clawed frog (*Xenopus tropicalis*)**	BCAP	XP_012822298	**Large yellow croaker (*Larimichthys crocea*)**	BCAP	XP_010732850
	BANK1	XP_031758988		BANK1	XP_027131322
**Elephant shark (*Callorhinchus milii*)**	BCAP	XP_007902749	**Greater amberjack (*Seriola dumerili*)**	BCAP	XP_022599643
	BANK1	XP_007890057		BANK1	XP_022614483
**Barramundi perch (Lates calcarifer)**	BCAP	XP_018536518	**Japanese lamprey (*Lampetra japonica*)**	BCAP	MW387160
	BANK1	XP_018536019		BANK1	-
**Sea lamprey (*Petromyzon marinus*)**	BCAP	AY152674	**Crown-of-thorns starfish (*Acanthaster planci*)**	BCAP	XP_022105714
	BANK1	XM_032969002		BANK1	-
**Pacific oyster Crassostrea gigas**	BCAP	XP_011419299	**Freshwater polyp Hydra vulgaris**	BCAP	-
	BANK1	-		BANK1	XP_047126799

Supplementary Table S2. The matching amino acid sequences in conserved motifs

Motif	Length (aa)	Best possible match
Motif 1	50	ELPTLLHFAAKYGLKNLTALLLQCPGALQAYSVANKYGDYPNTIAEKHGF
Motif 2	40	DPFAGMKTPGQRZLITLQEQVKLGIJSVDEAVEKFKZWQL
Motif 3	41	YSGDLIVAETQIEYYTDMEEIENLLANAABPVEFMCQAFKI
Motif 4	41	VPYNTETLDKLLTESLKKNIPASGLHLFGINQLEEEBMTTN
Motif 5	29	KRSESFRFQQENLKRLRDSITRRQKEKZK
Motif 6	28	VLQPPHKVVILLCGVEDSDILYELFPDW
Motif 7	21	DILIJYEEDAEEWALYLKSLF
Motif 8	29	PSVVVLPDRIRCGNPTEIFIJLKCEJDDE
Motif 9	21	RPSFIANRPPAPVPRPESSPP
Motif 10	38	DDSVYESMAHLSTDLLMKCSLNPGCDEELYESMAGFVP
Motif 11	50	FPPKKEJKRGDWKTESTSSTASSASNRSSTRSLLSVSSGMEGDSEDNEVP
Motif 12	29	KDLRQFIDEYVETADMLKSHIKEELMQGE
Motif 13	21	NEYTISVKAPDLPAGTVSVNV
Motif 14	50	GATEDLYVEMLQSKPDTPIARDEISLTTKDSMJRKFLEGNSMDMPDSEEG
Motif 15	34	YNLETSSLRRLELLSLSSYKCKLLILSNGLLECL
Motif 16	21	DNTPYISQVFQQKATRRPSDY
Motif 17	29	JSTDQEPEEYJSAVTKIISEGSGCDSETD
Motif 18	29	DLEITVPIRHSQNTPGKVECGIYEYGPRK
Motif 19	31	EDSISAZDLDLFQSSRCIVVLLSAELVZSFY
Motif 20	21	VKTEVEFSPENAPSVRVPAKL

Supplementary Table S3. List of primer sequences for gene cloning and qPCR

Gene	Primer	Sequences (5' to 3')	Application
Lja-BCAP	Forward	ATGGAAGGGACAGGCAGCTCT	ORF region amplification
	Reverse	TCATTGCCTGGGTGACTGTGG	
Lja-BCAP	Forward	CCGGAATTCATGGAAGGGACAGGCAGCTCT	Expression vector construction
	Reverse	CCCAAGCTTTCATTGCCTGGGTGACTGTGG	
Lja-BCAP	Forward	GTTCCGTCACCGTCCCAT	qPCR
	Reverse	GTCCTCCGCTTTTCCTTC	
gapdh	Forward	AACCAACTGCCTGGCTCCT	qPCR
	Reverse	GTCTTCTGCGTTGCCGTGT	

Supplementary Table S4. The predicted epitopes of Lja-BCAP

No.	Range	Peptides
1	5-14	LKAKQTCLRC
2	19-27	LDGTPLSRG
3	49-64	GLTDWPMTRDLLKLLV
4	79-83	EDAQG
5	89-93	HVSHR
6	117-152	GGTADELDSGMDSKTDTDTDTAAGDAGESSASPRGN
7	263-271	KIEPHDPER
8	284-307	LPASNVHLFGNHQLSNNNTQREEE
9	334-347	VQAYSVANRSGLYP
10	352-358	EQHGFQD
11	360-462	RKFMDDYVEAIIDENESEYMAMTPSRVASTPAPEDEEPYIAMGPGITEERSQQNLPKRASLKPT RSPPAVAQHQPEKGFEDASANQGPTRDHFAGMKTPGQQQ
12	471-476	KAGLIS
13	487-507	NQIDEKRRMASFKFHEENLKS
14	510-700	ESIARRKKEPSPRQPDLKEDIPVGLYESLPSKEVPFPSQGAFRHRPMRDSTSSTTSSVSSRSSTRS LQSLSSGAEADIEDGEGDMPHTMKEKRRTSELPMLEKKCVPPLPVGSKKMSSTSPTATAPSYP RPHSGSMTLQPPQIQHKAPPVTPRPPHTLSAKGREAPAAAGASAQPGGTPPPLLPRGKSPQS

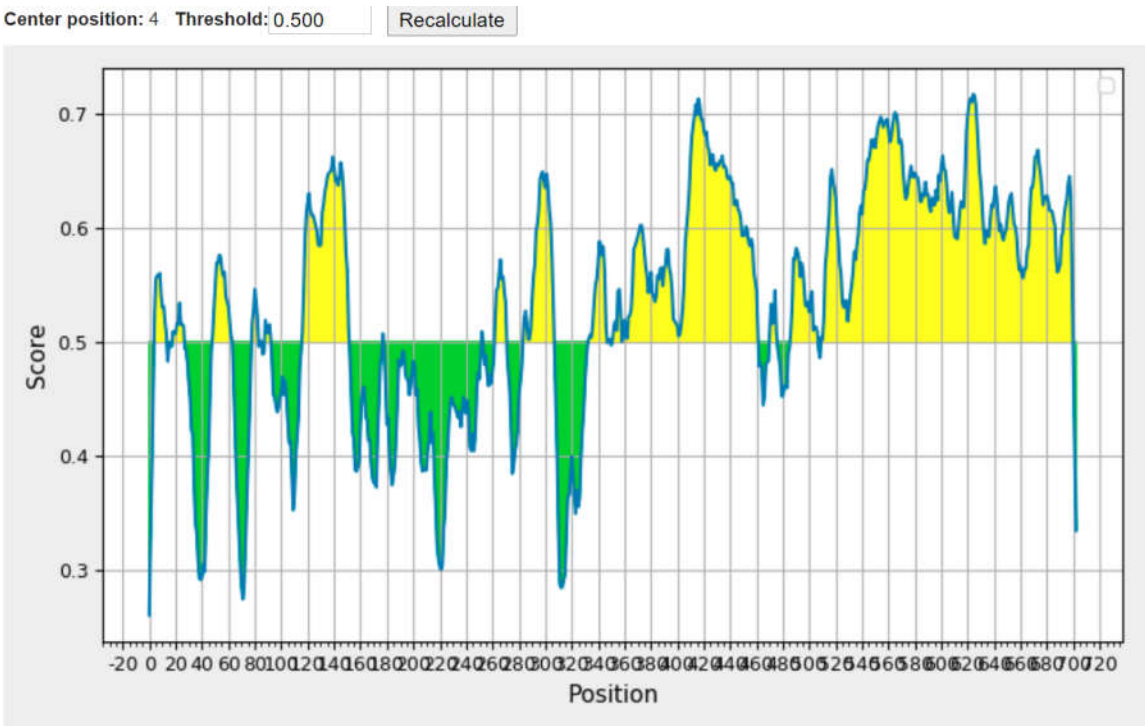

Supplementary Figure S1. The epitope prediction result of Lja-BCAP. The Bepipred Linear model was chosen for epitope prediction. Yellow indicates that the predicted score is beyond the threshold value.

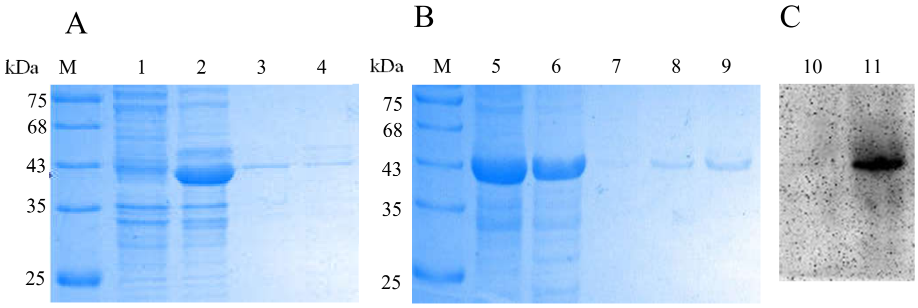

Supplementary Figure S2. The expression and purification of recombinant Lja-BCAP protein and the assay of the specificity of Lja-BCAP polyclonal antibody. **(A).** Recombinant expression and solubility test of truncated Lja-BCAP. M: Protein Marker; 1: Lysate of expression bacteria transformed with pET32a(+) plasmid (cultured at 30°C for 4 hours without induction by IPTG); 2: Lysate of expression bacteria transformed with Lja-BCAP-pET32a(+) recombinant plasmid (cultured at 30°C for 4 hours by adding 0.1 mmol/L IPTG induction); 3: Precipitation of lysate sample in Lane 3; 4: Supernatant of lysate sample in Lane 3. **(B).** Purification result of truncated Lja-BCAP recombinant protein. M: Protein Marker; 5: Expression bacteria lysate before purification; 6: Fraction flowed through the column; 7: Washing fraction; 8: 30mM imidazole buffer elution fraction; 9: Truncated Lja-BCAP recombinant protein after purification. **(C)**. Lja-BCAP polyclonal antibody specificity detection. 10 and 11: The prepared Lja-BCAP polyclonal antibody was used to detect the Lja-NICIR recombinant protein in the samples of lanes 1 (before induction) and 2 (after induction by 0.1 mmol/L IPTG), respectively.

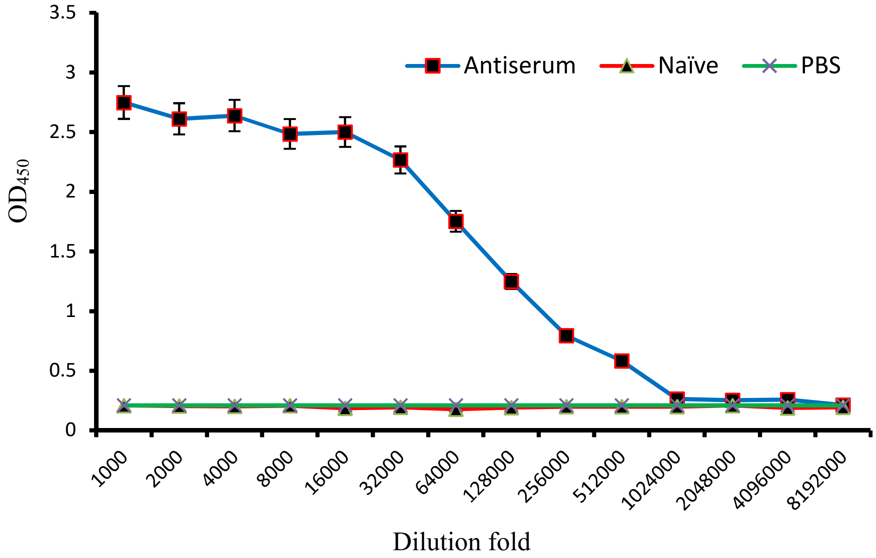

Supplementary Figure S3. The titer of Lja-BCAP polyclonal antibody detected by enzyme linked immunosorbent assay. The purified recombinant protein was used as antigen to coat the 96 well plate at a concentration of 2μg/ml. The antiserum of rabbit after the last immunization was tested as the first antibody. The antiserum was diluted according to the proportion shown in the abscissa in the figure. The horseradish peroxidase labeled sheep anti-rabbit IgG was used as the secondary antibody, and the OD450 value was determined by enzyme labeling instrument after the color was developed by tetramethylaniline solution. The antibody titer was determined when the OD450 value of the test hole was more than 2 times that of the negative control. The non-immunized rabbit serum (naïve) was taken as the negative control and PBS buffer as the blank control. Each sample was tested twice and their average value was taken as the results.

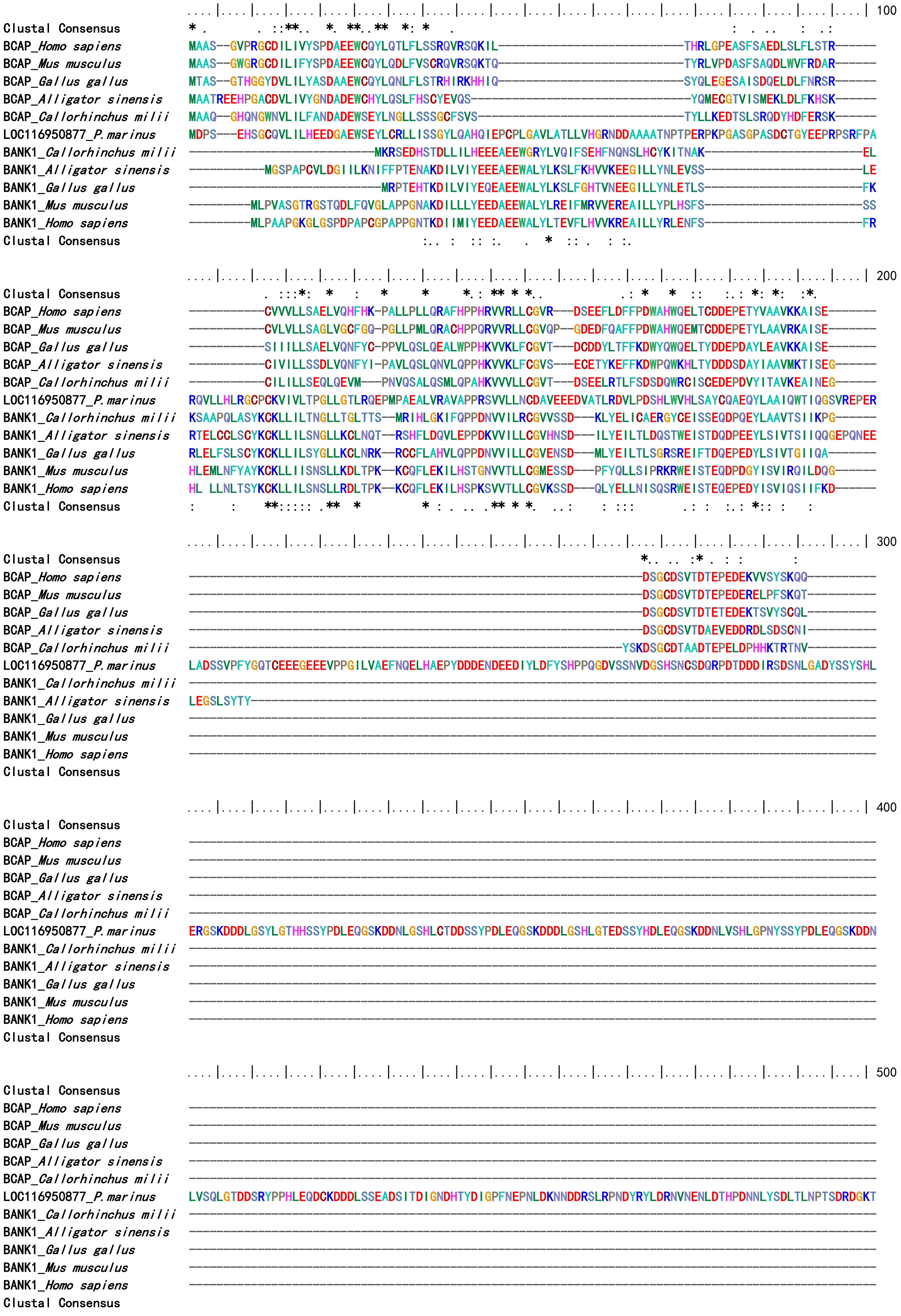
100
200
300
400
500
Clustal Consensus
BCAP_Homo sapiens
BCAP_Mus musculus
BCAP_Gallus gallus
BCAP_Alligator sinensis
BCAP_Callorhinchus milii
LOC116950877_P.marinus
BANK1_Callorhinchus milii
BANK1_Alligator sinensis
BANK1_Gallus gallus
BANK1_Mus musculus
BANK1_Homo sapiens
Clustal Consensus

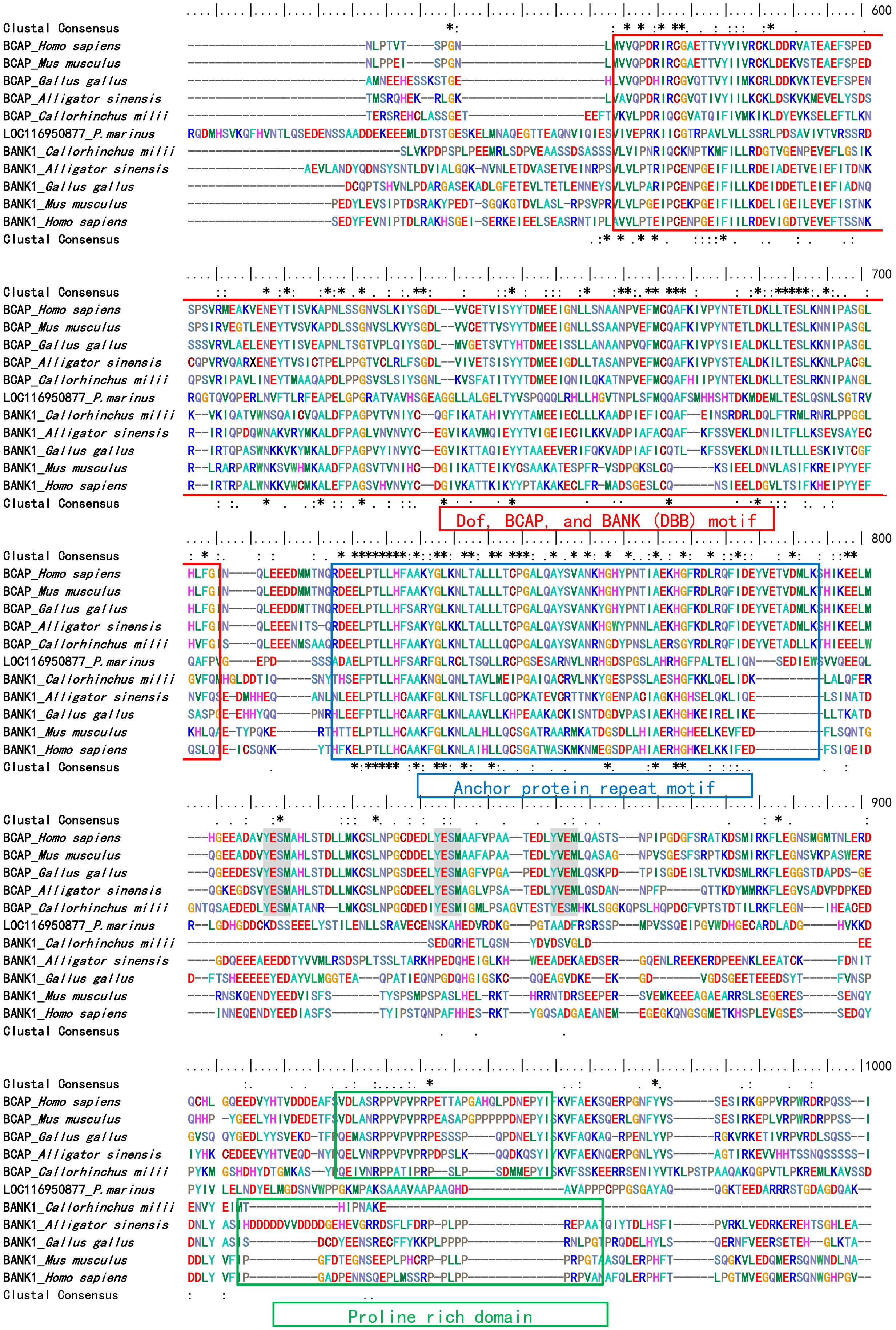
Clustal Consensus
BCAP_Homo sapiens
BCAP_Mus musculus
BCAP_Gallus gallus
BCAP_Alligator sinensis
BCAP_Callorhinchus milii
LOC116950877_P. marinus
BANK1_Callorhinchus milii
BANK1_Alligator sinensis
BANK1_Gallus gallus
BANK1_Mus musculus
BANK1_Homo sapiens
600
700
800
900
1000
Dof, BCAP, and BANK (DBB) motif
Anchor protein repeat motif
Proline rich domain

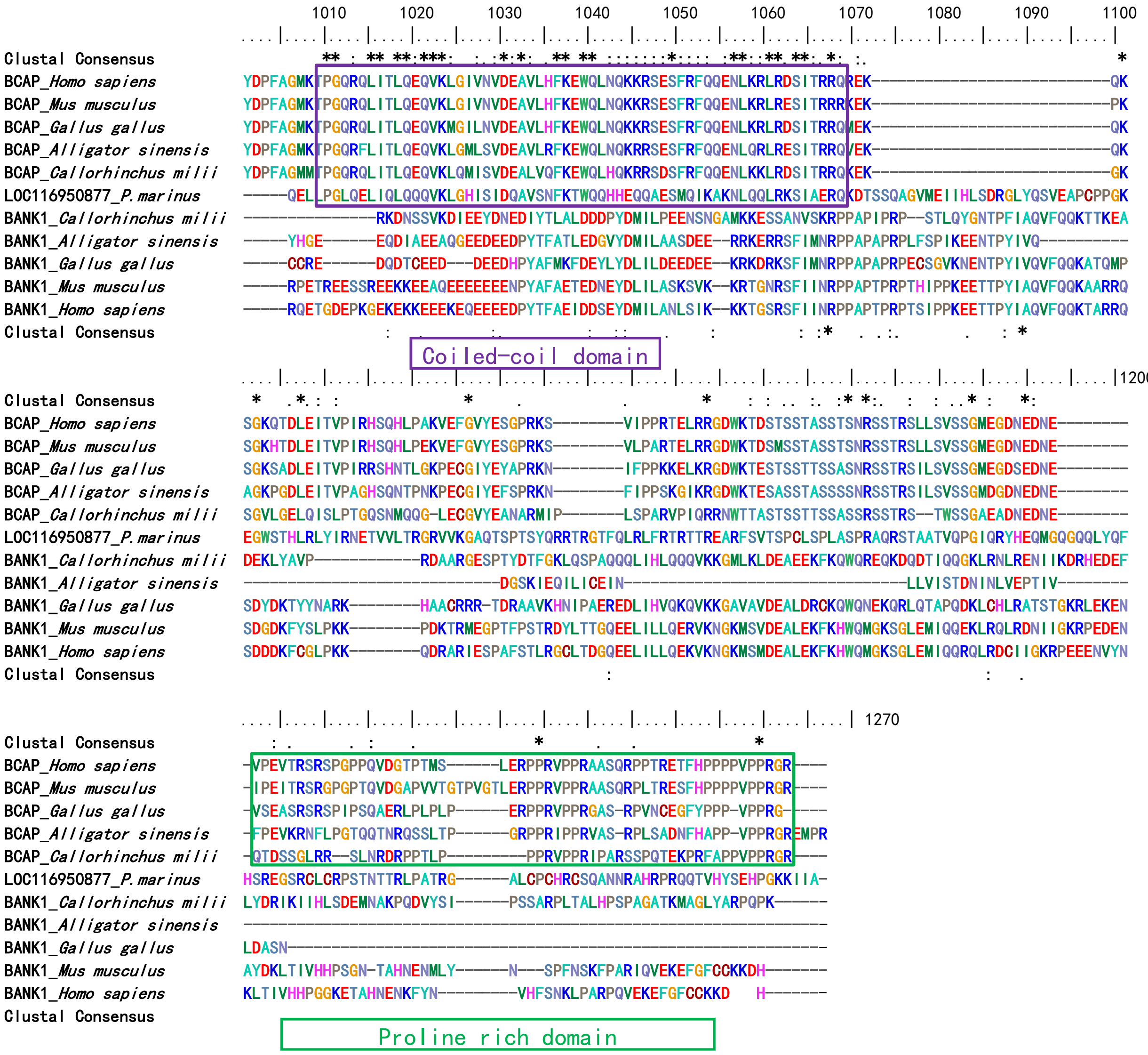

Supplementary Figure S4. Sequence alignment of uncharacterized LOC116950877 of *Petromyzon marinus* with several typical BCAP and BANK1 molecules by using Clustal X program. The accession numbers of the sequences are listed in Table S1. The identical amino acid residues among LOC116950877 and BCAP sequences are marked in the upper row of Clustal Consensus. The identical amino acid residues among LOC116950877 and BANK1 sequences are marked in the bottom row of Clustal Consensus. The functional domains such as Dof, BCAP, and BANK (DBB) motif, anchor protein repeat motif, proline rich domains and coiled-coil domain are marked by red, blue, green and purple frames, respectively. The conserved immunoreceptor tyrosine-based activation motifs are indicated by shaded letters.
